# General practice: a medical student perspective

**DOI:** 10.3402/meo.v20.28640

**Published:** 2015-09-03

**Authors:** Natalie Ramjeeawon, Neil Shah, Bharpoor Singh, Shawmian Singagireson

**Affiliations:** 1 University of Sheffield, Faculty of Medicine, Sheffield, UK; 2 Imperial College London, Faculty of Medicine, London, UK

In the run-up to the UK general election, all of the main political parties promised to increase the number of general practitioners (GPs) by 5,000–8,000, to curb ‘the crisis of the shortage of GPs’ (1). In 2013, only 20% of medical students opted for a career in general practice despite a national target of 50% by 2016 (2). So the question is, why are we not attracted to general practice? A study by the BBC cited a reason for this being the ‘declining status of the profession’ (2).

As a medical student, my first clinical experience was in a hospital, where I shadowed doctors and was left open to influence by their opinion. Any views held by these doctors had the potential to change my perception of differing career paths. I, along with most of my colleagues, have personally experienced the negative views of GPs held by some hospital doctors, especially regarding what they feel are inappropriate GP referrals to their specialist clinics. This undoubtedly could demolish one's aspiration for becoming a GP. These negative views could in part be fuelled by the current strain on the NHS, where specialist clinics are oversubscribed and specialists may thus become frustrated at times. There may also be unrealistic expectations of GPs by specialists, expecting a greater level of knowledge in their subspecialty, whereas GPs are by definition generalists.

To counteract this, I feel that earlier exposure to general practice for all medical students, with an emphasis on independent consultations with patients and designated teaching time, is needed. A better understanding of the work that GPs do at earlier stages of our medical career would be beneficial in realising the pivotal and diverse role they have in primary care. In addition, understanding difficulties GPs face on a daily basis of reassuring patients whilst still ensuring appropriate hospital referrals may prevent negativity in secondary care. Many medical schools have implemented earlier general practice attachments in the first 2 years for this reason.

The ‘crisis’ is not one to be ignored. With a profound mismatch between supply and demand, any positive promotion of a GP career in the early stages of medical education should be implemented rapidly. Only then can we provide the ageing and growing UK population good access to primary care. This is fundamental for the ‘cost-effective, high-quality NHS that meets the needs of the population’ (3).
